# Entropy Generation Analysis of a Heat Exchanger for Precision Applications

**DOI:** 10.3390/e28080867

**Published:** 2026-08-01

**Authors:** Maria Valeria De Bonis, Diego Alcañiz, Paolo Caccavale, Gianpaolo Ruocco

**Affiliations:** 1 Initiatives for Bio-Materials Behaviour Srls, University of Basilicata, 85100 Potenza, Italy; mv.debonis@gmail.com; 2 Department of Engineering, University of Basilicata, 85100 Potenza, Italy; diealcos@gmail.com; 3 Fluere, 25124 Brescia, Italy; paolo.caccavale@fluere.it; 4 Institute for Polymers, Composites and Biomaterials, National Council of Research, 80078 Pozzuoli, Italy

**Keywords:** entropy generation, computational fluid dynamics, process intensification

## Abstract

Process heating for sensitive fluids requires a sophisticated balance of efficiency and thermal control. This study assesses the efficiency of a singular active-surface flow box delivering high-precision, volumetric energy transport. Entropy generation analysis coupled with conjugate heat transfer can be effectively conducted using three-dimensional CFD to monitor the local irreversibilities stemming from direct contact with the active surface (heat transfer) and macro-steady flow structures (viscous dissipation). The entropy generation metric proposed, based on a Brinkman number, demonstrates that the global entropy generated grows less than linearly with the applied thermal power, while only a slight decrease is found with the increasing flow rate, for the two fluids examined. We conclude that integrating entropy generation analysis within a CFD framework is a robust tool for optimizing heaters for precision processing. For sensitive fluids such as liquid foods, this methodology is particularly valuable, as it directly links thermodynamic efficiency to quality retention by limiting excessive local temperatures. This research advances the development of entropy-informed thermo-fluid systems, offering a clear pathway for the design of high-performance, precision heating devices.

## 1. Introduction

Industrial process heat is undergoing a radical transformation as electrification becomes the cornerstone of decarbonization. Driven by the global expansion of renewable sources, electricity-driven heating offers a carbon-neutral alternative to conventional fossil fuels [1,2]. However, the inherent intermittency of these sources remains a significant hurdle, making large-scale industrial integration a critical challenge [3].

To process sensitive liquids without compromising quality, the industry is shifting toward precision technologies such as microwave-assisted heating [4], ohmic heating [5], and high-pressure processing [6]. Within this framework, consolidating energy transport into a single, high-inertia ceramic plate eliminates thermal noise and enforces a strictly unidirectional heat flux [7,8]. When the active plate is fed by volumetric microwave heating, the micrometric or Precision Flow Box Heat EXchanger (PFB-HEX) achieves high-density heat transfer that combines exceptional efficiency with the precise thermal control required for sensitive fluids. Bridging the operating characteristics of channel flows and jet loop reactors [9,10], such a device ensures operating precision and flexibility. Our previous research [8] established that these flow structures govern local energy distribution and mixing—the critical determinants of heat transfer performance when processing sensitive fluids. The present work represents a Second Law approach of such research, developing new metrics useful to pursue process efficiency.

Since the seminal work of Bejan [11], entropy generation has served as the definitive metric for quantifying process irreversibilities. By employing Entropy Generation Minimization (EGM) [12], designers can rigorously evaluate competing configurations to isolate optimal parameters that maximize energy efficiency. This approach transforms entropy from a theoretical abstraction into a precise diagnostic tool for engineering high-performance thermal systems.

To optimize the PFB-HEX, we must transition from global thermodynamic balances to localized analysis. Hesselgreaves [13] first employed EGM analysis for HEXs to show that a rational EGM method leading to HEX optimization exists. Then, EGM analysis was updated and extended to many other HEX configurations by Manjunath and Kaushik [14] analyzing fluid flow, mostly in laminar regimes. Zhang et al. [15] dealt with counterflow, parallel flow, crossflow or any other HEX configuration correlating EGM and flow rate, showing that the higher the temperature difference and the lower the fluid flow, the lower the entropy generated, while Zheng et al. [16] reviewed the single-phase convective heat transfer enhancement based on multi-longitudinal vortices in HEX tubes.

However, traditional EGM often overlooks the impact of macro-steady flow structures—such as those found in the PFB-HEX—which fundamentally alter thermal processing. Local entropy generation can be studied by the simulation of convection heat transfer that uses the governing equations of entropy generation which correlate with such dependent variables as studied in jet impingement heat transfer [17,18] and for a finned-tube heat exchanger [19]. Computational Fluid Dynamics (CFD) was employed by Kock and Herwig [20], Ji et al. [21] to compute the local entropy generation in non-isothermal turbulent flows. The topic was then reviewed by [22], while more recently, [23] used the CFD to compare the entropy generated by a variety of HEX configurations and cross sections, relying greatly on the average Reynolds number when formulating design recommendations. By integrating custom entropy generation equations directly into commercial multiphysics environments [24], we can treat entropy as a primary governing variable, transforming it into a powerful diagnostic for optimizing precision heat transfer.

The present work is aimed at entropy generation analysis in a PFB-HEX prototype by pointing at the optimal design and operating parameters when an all-purpose CFD tool is effectively employed. The flow-box geometry induces macro-steady structures that “nudge” the fluid into controlled jet-loops, sweeping the active plate at optimal velocity for maximum uptake with minimal residence time. This singular interface makes then entropy generation fully resolvable, enabling direct identification of hotspots, precise mixing control, and systematic minimization of irreversibility. The result is a heat exchanger where transfer becomes a controlled event—combining macroscopic thermal inertia with micro-reactor precision for renewable-ready processing. The exploitation of entropy generation calculations within a general CFD tool helps understand different active surface configurations and/or the implementation of modified contact surfaces to promote mixing and hence induce more uniform and quality-preserving flow patterns, as sensitive fluids need to be protected from excessive, local overheating.

## 2. Problem Formulation

**Figure 1:**
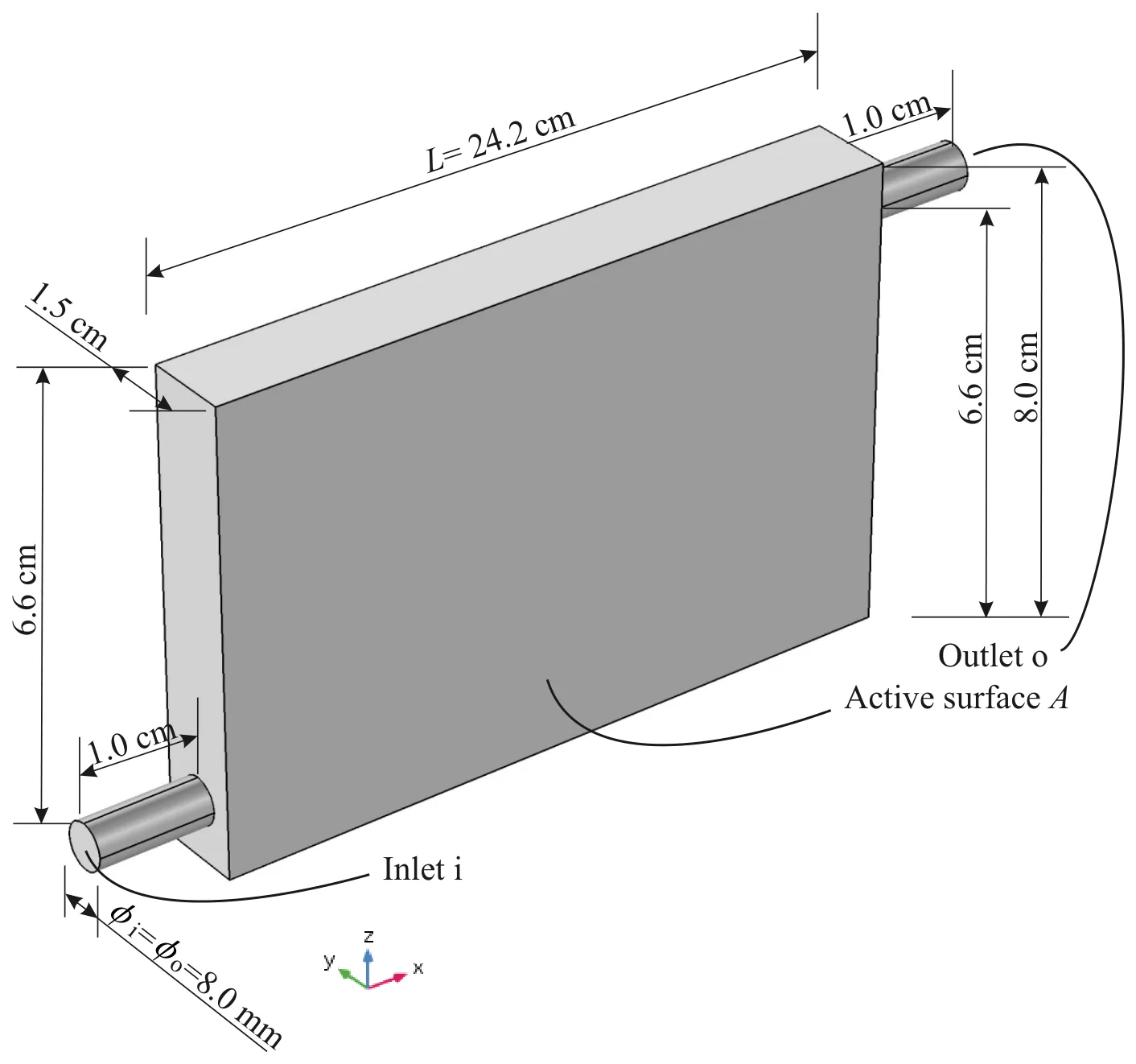
The PFB-HEX flow box under scrutiny, with indication of the active surface area *A*, and inlet and outlet sections *i* and *o*.

In the present work we investigate the entropy generation of a precision fluid heater, consisting of two slender-shaped flow boxes or control volumes (CVs), heated by an interspersed active PFB-HEX plate. In Figure 1, one such CV is depicted, including the annexed active plate. This CV is connected by means of a 8 mm diameter branch of adequate length, to allow flow development, to a hold-up tank and circulation pump.

PFB-HEX heating is operated by exploiting the ceramic plate properties, i.e., rapidly accumulating heat at before the actual flow circulation, then releasing during flow contact. The goal is to protect heat-sensitive fluids by undesired temperature extremes (both overheating and underheating) to ensure proper quality/safety processing (thermization). Details on the experimental rig and PFB-HEX development are reported in the companion papers [7,8].

### 2.1. Driving Assumptions

Based on the circulated operation described above, and the small characteristic length of the heat transfer geometry of the heater at stake (available flow volume divided by active heat transfer surface), a steady-state regime is assumed: the flow field (after a brief start-up transient) does not change during the process, and a constant temperature field is established in the fluid.

The following additional assumptions are adopted:

1. The flow is incompressible (negligible pressure work and kinetic energy) with temperature-dependent properties. Due to the adopted flow regime, as later implied, no body force is accounted for.
2. The viscous heat dissipation is neglected.
3. All non-active wall surfaces are adiabatic.
4. No-slip is enforced at every solid surface.

### 2.2. Governing Equations

With reference to the previous statements, and supplementing what was brought forth already in [8], it is recognized that the flow reaches statistical equilibrium quickly, while the thermal inertia evolves over longer timescales. Therefore, the thermo-fluid behavior within the flow box is described through a steady-state incompressible Reynolds-Averaged Navier–Stokes (RANS) formulation coupled with the low-Reynolds k−ε turbulence model, and the transient energy equation [25], to yield the mean fluid velocity and instantaneous temperature, when the Reynolds decomposition is applied to the dependent variables, as appropriate.

*Flow continuity and Momentum transfer:*

(1) $$\nabla \cdot \bar{v}=0\;;\;\rho \bar{v}\cdot \nabla \bar{v}=-\nabla \bar{p}+\nabla \cdot \left[\left(\mu +\mu_{t}\right)\left(\nabla \bar{v}+\nabla {\bar{v}}^{T}\right)\right]$$

where $\mu_{t}$ is the turbulent (eddy) viscosity evaluated through the low-Reynolds k−ε closure model, computed as

(2) $$\mu_{t}=\rho C_{\mu }f_{\mu }\frac{k^{2}}{\varepsilon }$$

with *k* as the turbulent kinetic energy, ε as the turbulent dissipation rate, $C_{\mu }$ as a model constant, and $f_{\mu }$ as the low-Reynolds damping function [26]. Moreover, g is the gravitational acceleration vector, and β is the thermal expansion coefficient.

*Transport of turbulent kinetic energy*:

(3) $$\rho \bar{v}\cdot \nabla k=\nabla \cdot \left[\left(\mu +\frac{\mu_{t}}{\sigma_{k}}\right)\nabla k\right]+P_{k}-\rho \varepsilon$$

*Transport of turbulent dissipation rate*:

(4) $$\rho \bar{v}\cdot \nabla \varepsilon =\nabla \cdot \left[\left(\mu +\frac{\mu_{t}}{\sigma_{\varepsilon }}\right)\nabla \varepsilon \right]+C_{\varepsilon 1}f_{1}\frac{\varepsilon }{k}P_{k}-C_{\varepsilon 2}f_{2}\rho \frac{\varepsilon^{2}}{k}$$

where $P_{k}$ is the turbulence production term and $f_{1},f_{2}$ are additional low-Reynolds correction functions [26].

*Transfer of energy*:

(5) $$\rho c_{p}\frac{\partial \bar{T}\left(t\right)}{\partial t}+\rho c_{p}\bar{v}\cdot \nabla \bar{T}\left(t\right)=\nabla \cdot \left[\left(\lambda +\lambda_{t}\right)\nabla \bar{T}\left(t\right)\right]$$

where the dependence of temperature on time is emphasized, and the turbulent thermal conductivity is evaluated as

(6) $$\lambda_{t}=\frac{\mu_{t}c_{p}}{{\mathrm{Pr}}_{t}}$$

with ${\mathrm{Pr}}_{t}$ as the turbulent Prandtl number, assumed equal to 1.0 as adequate for confined thermal flows with strong momentum–thermal coupling.

The governing equations, with standard constants in Equations (2)–(4), are solved through COMSOL Multiphysics^®^ [24].

### 2.3. Initial and Boundary Conditions

With reference to Figure 1, the following boundary conditions are imposed:

- Initial condition for temperature: $\bar{T}=T_{i}$
- Given conditions at inlet i: ${\bar{v}}_{x}=v_{i},\;{\bar{v}}_{y}=0,\;{\bar{v}}_{z}=0,\;\bar{T}=T_{i}$ Turbulence quantities at the inlet are prescribed through turbulence intensity and length scale relationships.
- At outlet o: $\frac{\partial {\bar{v}}_{x}}{\partial x}=0,\;{\bar{v}}_{y}={\bar{v}}_{z}=0,\;\bar{p}=0,\;\nabla \bar{T}\cdot n=0$
- No-slip condition at all flow-box walls: $\bar{v}=0$. Low-Reynolds wall treatment is intrinsically resolved through the turbulence model without wall functions.
- Adiabatic condition at all flow-box walls except at the active surface *A*: $-n\cdot \lambda \nabla \bar{T}=0$
- Prescribed heat flux at the active surface *A*: $-n\cdot \lambda \nabla \bar{T}=\dot{q}$

### 2.4. Entropy Generation for Process Flow

The local volumetric entropy generation rate is evaluated according to the second-law formulation for convective thermo-fluid processes proposed by Bejan [12], accounting for irreversibilities associated with finite temperature gradients and viscous dissipation within a Reynolds-averaged framework. The total local entropy generation rate can be decomposed as

(7) $${\dot{S}}_{\mathrm{gen}}^{\prime \prime \prime }={\dot{S}}_{\mathrm{ht}}^{\prime \prime \prime }+{\dot{S}}_{\mathrm{ff}}^{\prime \prime \prime }$$

where the thermal and fluid-friction contributions respectively read:

(8) $${\dot{S}}_{\mathrm{ht}}^{\prime \prime \prime }=\frac{\lambda_{\mathrm{eff}}}{{\bar{T}}^{\;2}}\left[{\left(\frac{\partial \bar{T}}{\partial x}\right)}^{2}+{\left(\frac{\partial \bar{T}}{\partial y}\right)}^{2}+{\left(\frac{\partial \bar{T}}{\partial z}\right)}^{2}\right]$$

and

(9) $$\begin{array}{cc} {\dot{S}}_{\mathrm{ff}}^{\prime \prime \prime }=\frac{\mu_{\mathrm{eff}}}{\bar{T}}\{\; & 2\left[{\left(\frac{\partial {\bar{v}}_{x}}{\partial x}\right)}^{2}+{\left(\frac{\partial {\bar{v}}_{y}}{\partial y}\right)}^{2}+{\left(\frac{\partial {\bar{v}}_{z}}{\partial z}\right)}^{2}\right]+\\ \; & {\left(\frac{\partial {\bar{v}}_{x}}{\partial y}+\frac{\partial {\bar{v}}_{y}}{\partial x}\right)}^{2}+{\left(\frac{\partial {\bar{v}}_{x}}{\partial z}+\frac{\partial {\bar{v}}_{z}}{\partial x}\right)}^{2}+{\left(\frac{\partial {\bar{v}}_{y}}{\partial z}+\frac{\partial {\bar{v}}_{z}}{\partial y}\right)}^{2}\} \end{array}$$

In accordance with the RANS-based entropy generation framework, the effective transport properties are defined as

(10) $$\mu_{\mathrm{eff}}=\mu +\mu_{t}\;;\;\lambda_{\mathrm{eff}}=\lambda +\lambda_{t}$$

The local absolute temperature field $\bar{T}$, rather than the inlet temperature, is employed in the entropy-generation terms consistently with the local formulation of irreversible thermodynamics, allowing accurate spatial resolution of irreversibility sources associated with thermal gradients and viscous dissipation.

The total entropy generation rate within the flow domain is finally obtained through volumetric integration:

(11) $${\dot{S}}_{\mathrm{gen}}={\dot{S}}_{\mathrm{ht}}+{\dot{S}}_{\mathrm{ff}}=\int_{V}{\dot{S}}_{\mathrm{ht}}^{\prime \prime \prime }\;dV+\int_{V}{\dot{S}}_{\mathrm{ff}}^{\prime \prime \prime }\;dV$$

### 2.5. Numerical Treatment

In all calculations, an adequate inlet spout has now been provided, with respect to the original geometry [8], to ensure complete development of turbulent channel flow. Integration of the partial differential Equations (1)–(5), along with their boundary conditions, was carried out by employing first the direct MUMPS solver for the wall distance initialization, with the automatic preordering algorithm and a relative tolerance of $1\times 10^{-3}$; then the direct PARDISO solver with nested multithreaded dissection and a relative tolerance of $1\times 10^{-4}$ was invoked in a segregated fashion with row preordering and multithreaded forward and backward solve, to ensure computational stability and robustness: first for velocity and pressure, then for temperature. Finally, dedicated calculations were executed after convergence for Equations (8), (9) and (11).

**Figure 2:**
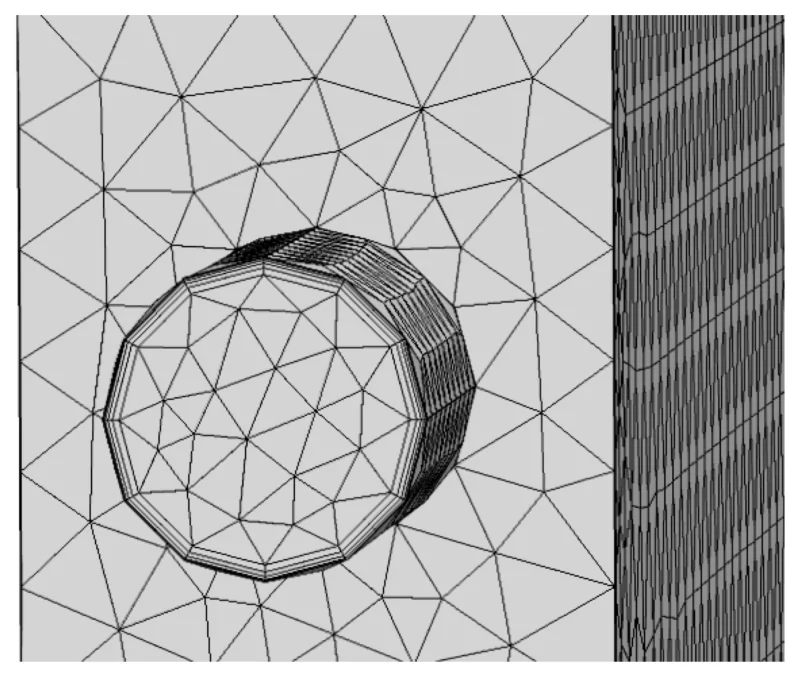
Close-up of the employed finite element grid of Figure 1, featuring tetrahedral and boundary layer cells, before the flow box inlet. This kind of grid was adopted along all solid walls in the CV.

A tetrahedral grid of $109\times 10^{3}$ total cells was devised for the PFB-HEX geometry as in Figure 1, featuring a boundary layer grid of 4 levels having a thickness of $5\times 10^{-5}$ m, which allowed for resolving the velocity and temperature gradients in the boundary layer, along all solid walls (as an example, see Figure 2). A grid independency test was carefully performed prior to the generation of results by a recursive refinement for both the total cell number and the number and thickness of boundary layer grid levels up to $148\times 10^{3}$ total cells, with a positive check on the averaged fluid temperature at outlet $T_{o}$ (whereas, for the sake of simplicity, the averaging notation is omitted). In particular, an invariance up to the 4th significant digit was sought on a $122\times 10^{3}$ total cells domain, carrying a boundary layer grid of 5 levels having a thickness of $3\times 10^{-5}$ m. The computing time of each run took about 0.5 h by using a Intel Xeon server (Windows 10 OS, eight-core, 32-thread at 2.4 GHz, 128 GB RAM).

## 3. Results

### 3.1. Parameter Space and Reduction

For the PFB-HEX geometry in Figure 1, the governing parameters are the power applied to the active plate $\dot{q}A$ (from 150 to 400 W), the inlet volume flow rate $\dot{V}$ (from 1.00 to 8.00 L/min), and the fluid type (water or common sunflower oil). For the sake of simplicity, assuming a prevalent x-wise transport, a conventional hydraulic diameter $D_{h}$ is calculated based on the related wetted cross-sectional area Ω=12 cm^2^ and its wetted perimeter so that $D_{h}\approx 2.53$ cm. Across the range of flow rates, transitional or turbulent regimes are established for both oil and water (with inlet Reynolds numbers up to about $4\times 10^{2}$ for oil, and $2\times 10^{4}$ for water). A base case is configured for $\dot{V}=2.00$ L/min of water warmed by $\dot{q}A=200$ W, and inlet temperature $T_{i}$ kept always at 294 K.

Parameter reduction for a proper entropy-regime characterization framework must be performed to account for thermal and hydrodynamic forcing, irreversibility contributions and global budget. Firstly, the bulk HEX behavior can be described based on a suitable Brinkman number containing the viscous, inertia and thermal scales [27,28,29]:

(12) $$\mathrm{Br}=\frac{\mu \left(T_{i}\right){\left|v_{\Omega }\right|}^{2}}{\lambda \left(T_{i}\right)\left(T_{o}-T_{i}\right)}$$

Then, the entropy generation analysis the can be finalized through the Bejan number and a global entropy generation number $N_{\dot{S}}$:

(13) $$\;\mathrm{Be}=\frac{{\dot{S}}_{\mathrm{ht}}}{{\dot{S}}_{\mathrm{ht}}+{\dot{S}}_{\mathrm{ff}}}\;;\;N_{\dot{S}}=\frac{{\dot{S}}_{\mathrm{gen}}T_{i}}{\dot{q}A}$$

### 3.2. Thermization, Flow Patterns, and Local Entropy Distributions

The agreement between the model results and the experiments was presented in the accompanying paper [8]: for a base case of water flowing with $\dot{V}=2.00$ L/min, and heated by $\dot{q}A=200$ W, the model gave a computed temperature increment within an error of about 5% with respect to the above experimental measurements.

**Figure 3:**
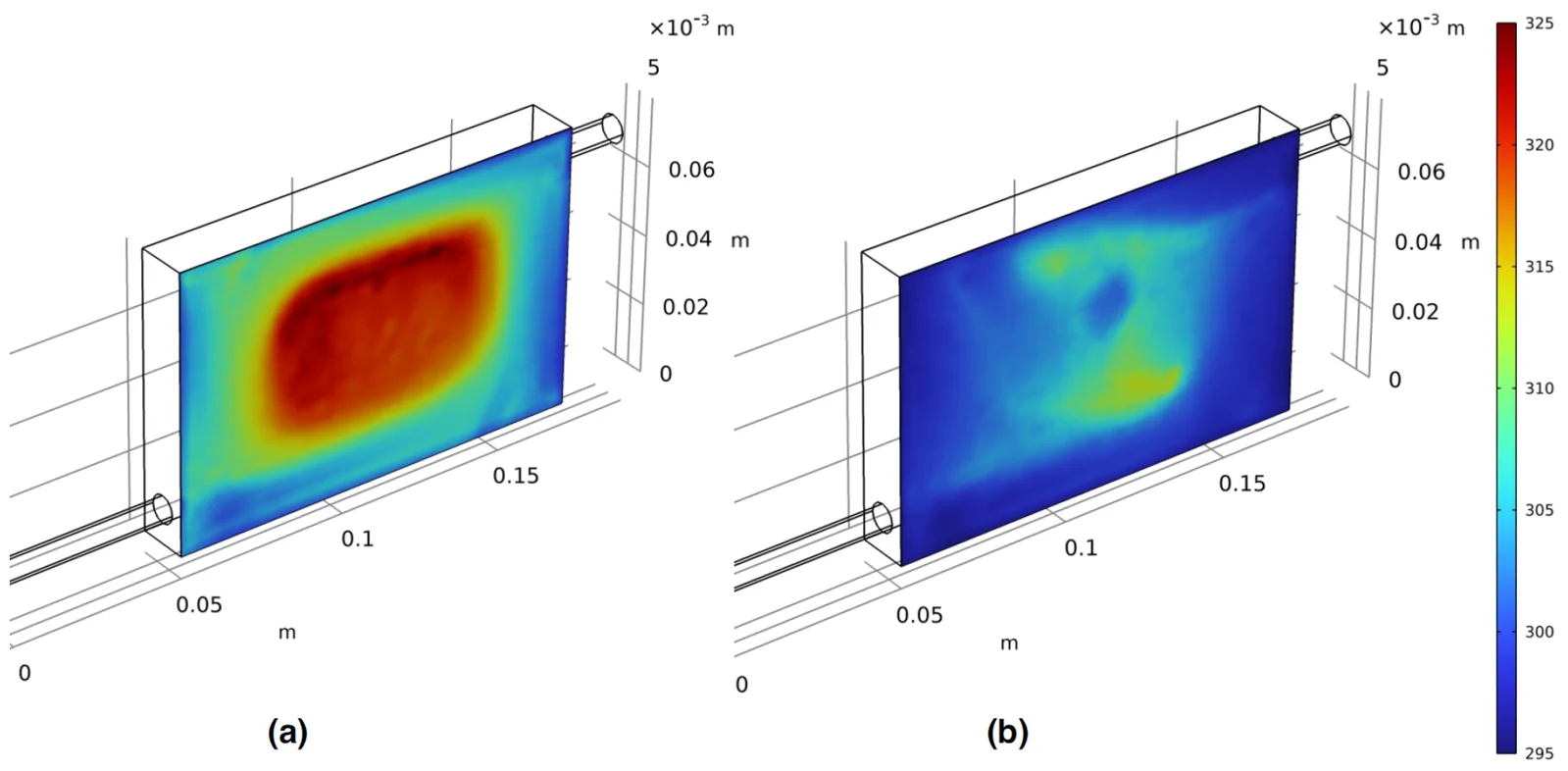
Steady-state distribution (after 4 min of operation) of $\bar{T}$ (K) in a fluid section in direct contact to active surface *A* for the base case and two values of the volume flow rate $\dot{V}$. (**a**) 2.00 L/min. (**b**) 8.00 L/min.

**Figure 4:**
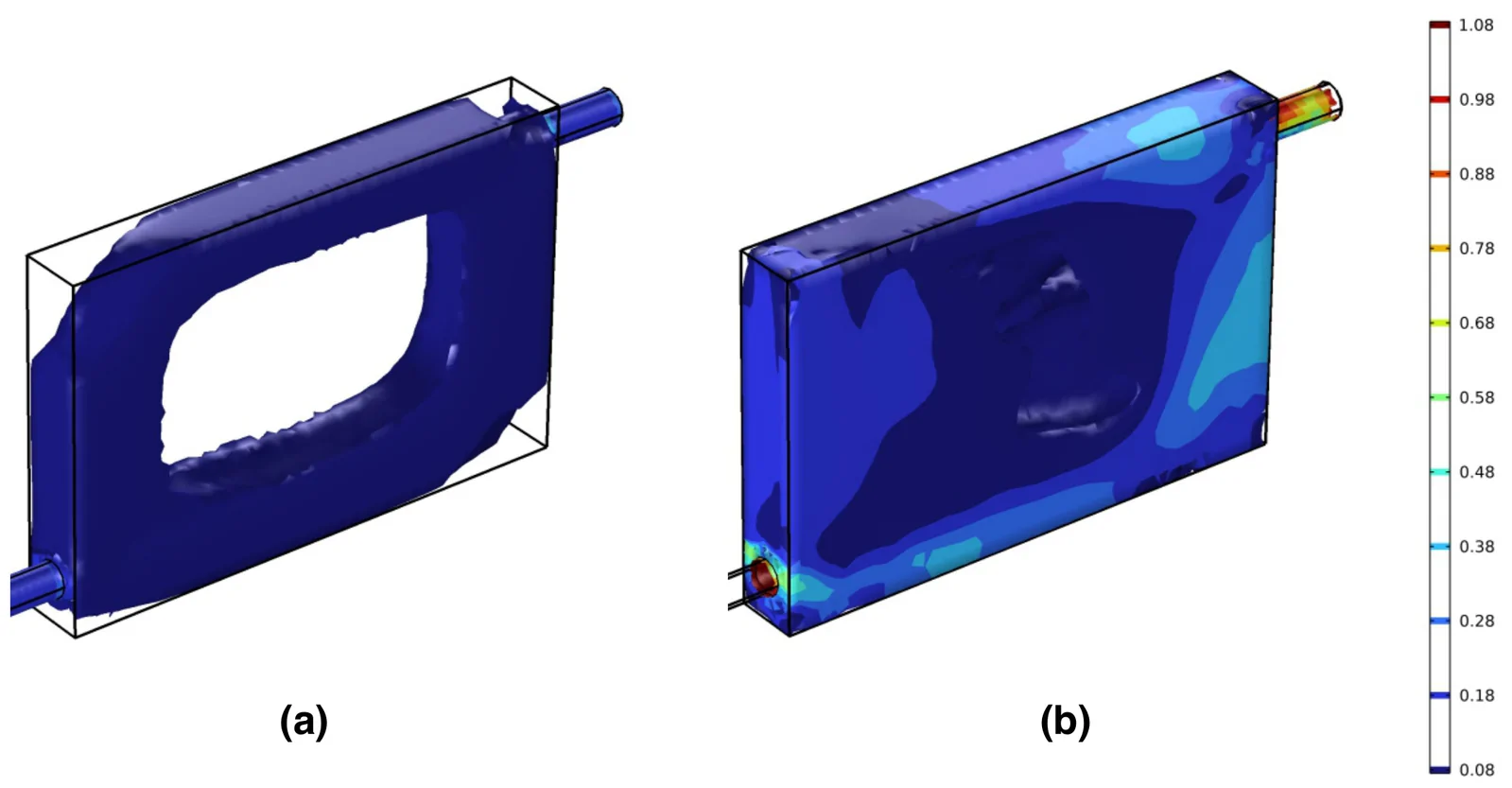
Steady-state representation (after 4 min of operation) of isosurface $\left|\bar{v}\right|$ (m/s) for the base case and two values of the volume flow rate $\dot{V}$. (**a**) 2.00 L/min. (**b**) 8.00 L/min.

Different thermal distributions and flow patterns are obtained when exploring the variable space, as reported for example in Figure 3 and Figure 4, respectively, for a variation of $\dot{V}$.

**Figure 5:**
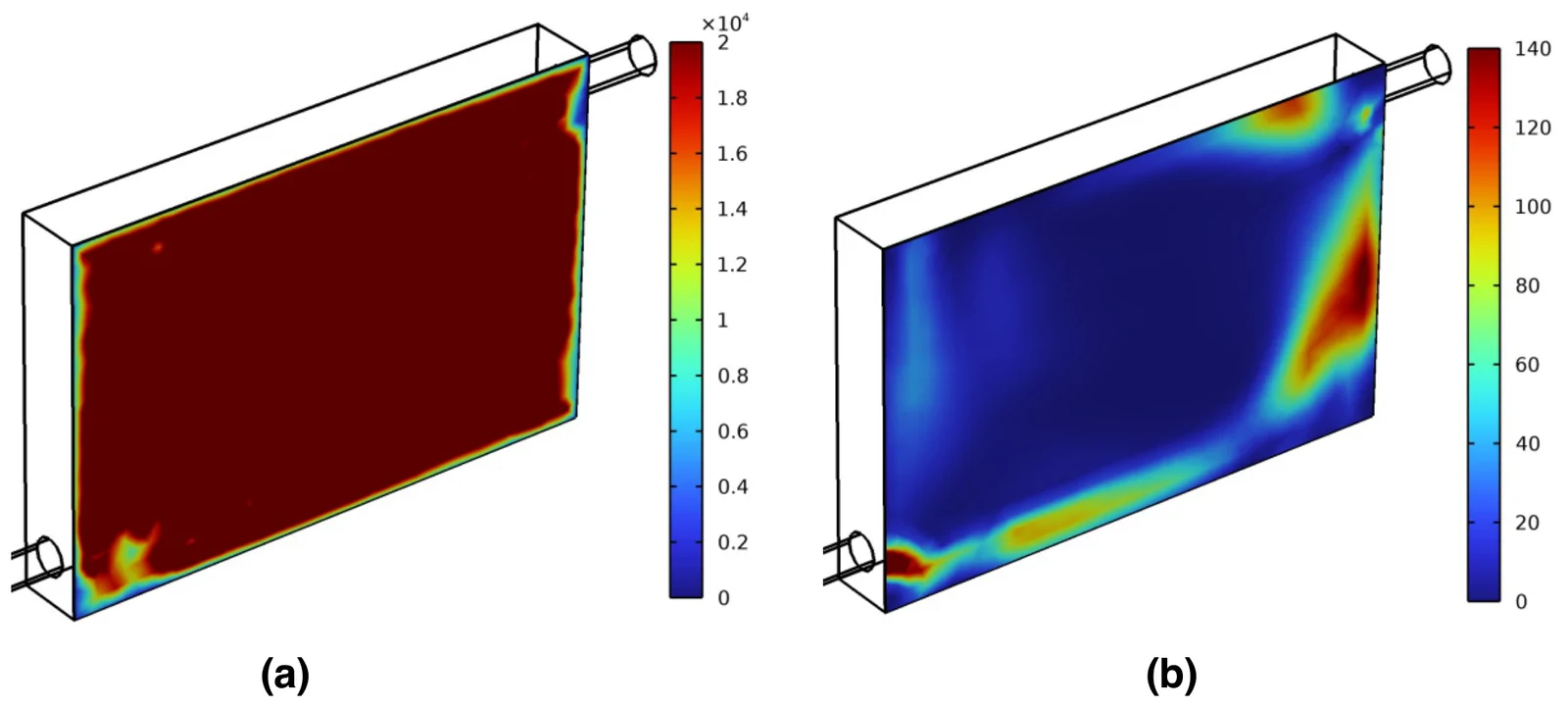
Steady-state distribution of ${\dot{S}}_{\mathrm{ht}}^{\prime \prime \prime }$ and ${\dot{S}}_{\mathrm{ff}}^{\prime \prime \prime }$ (W m^−3^K^−1^) contributions in a fluid section in direct contact to active surface *A* for water, $\dot{q}A=400$ W and $\dot{V}=8.00$ L/min. (**a**) ${\dot{S}}_{\mathrm{ht}}^{\prime \prime \prime }$. (**b**) ${\dot{S}}_{\mathrm{ff}}^{\prime \prime \prime }$.

Consequently, it is expected that, depending on the operating parameters, high-temperature gradients and recirculation patterns impinge upon the computation of the local entropy generation rate. The two contributions of local irreversibility are therefore depicted in Figure 5, where an example of operation is reported.

### 3.3. An Entropy Generation-Regime Chart

**Figure 6:**
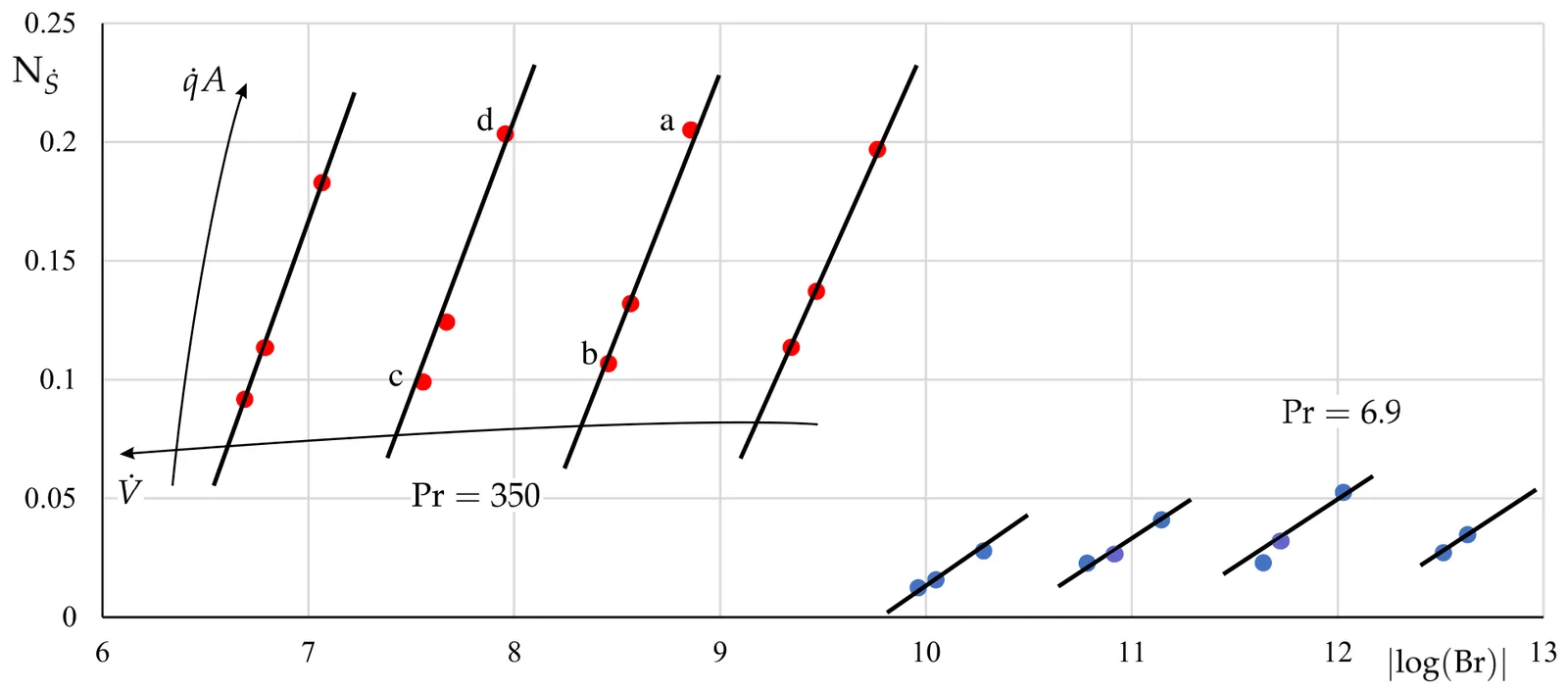
An entropy generation-regime chart, reporting on $N_{\dot{S}}=f\left(\mathrm{Br}\right)$ for two values of the Prandtl number. The varying direction of the forcing parameters $\dot{q}A$ and $\dot{V}$ are indicated.

The numerical experiments in the considered parameter space have been carried out to come up with the entropy generation-regime $N_{\dot{S}}=f\left(\mathrm{Br}\right)$ chart of Figure 6, reporting on two groups of similarity segments (for each value of the Prandtl number). In each group, the two forcing parameters (i.e., the applied power and volume flow rate) vary as indicated.

### 3.4. Transient Behavior of the Modified PFB-HEX

**Figure 7:**
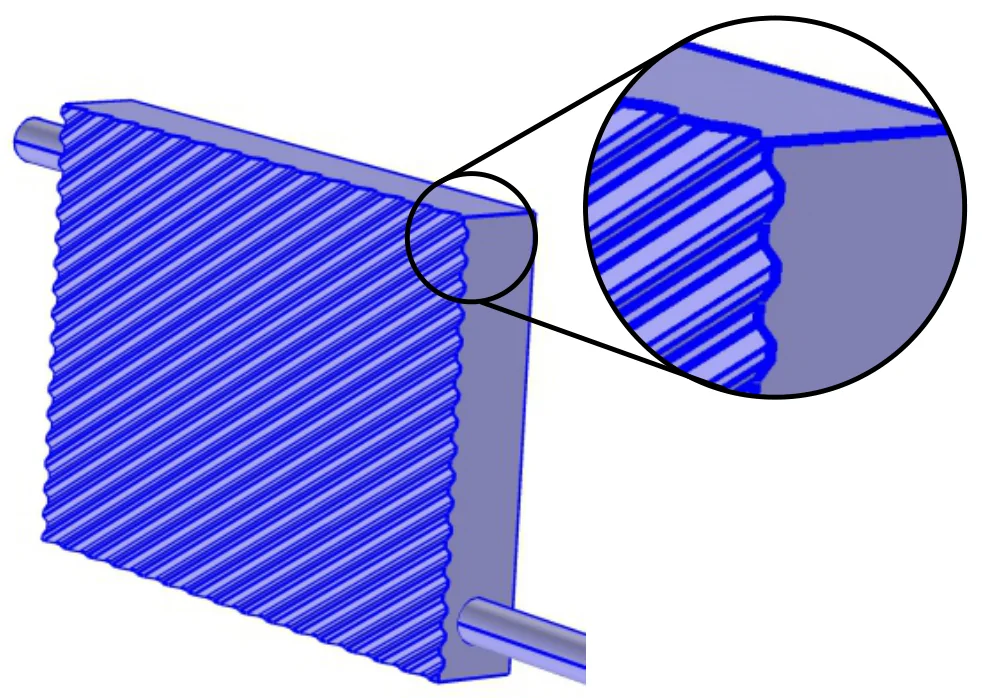
Active surface *A* preliminarily modified by 30°-inclined grooves (as depicted in the inset), etched to form a repeating sequence of isosceles trapezoidal features, each 1 mm high with bases of 3 and 1 mm.

In this work, a straightforward yet preliminary modification of the active thermal surface *A* is attempted, without further alteration of the overall PFB-HEX geometry. To this end, diagonally arranged grooves are introduced on *A* (as shown in Figure 7), to promote localized flow deceleration and repeated boundary-layer redevelopment in the vicinity of the thermally active interface so as to increase the effective fluid residence time within the higher temperature confinement.

**Figure 8:**
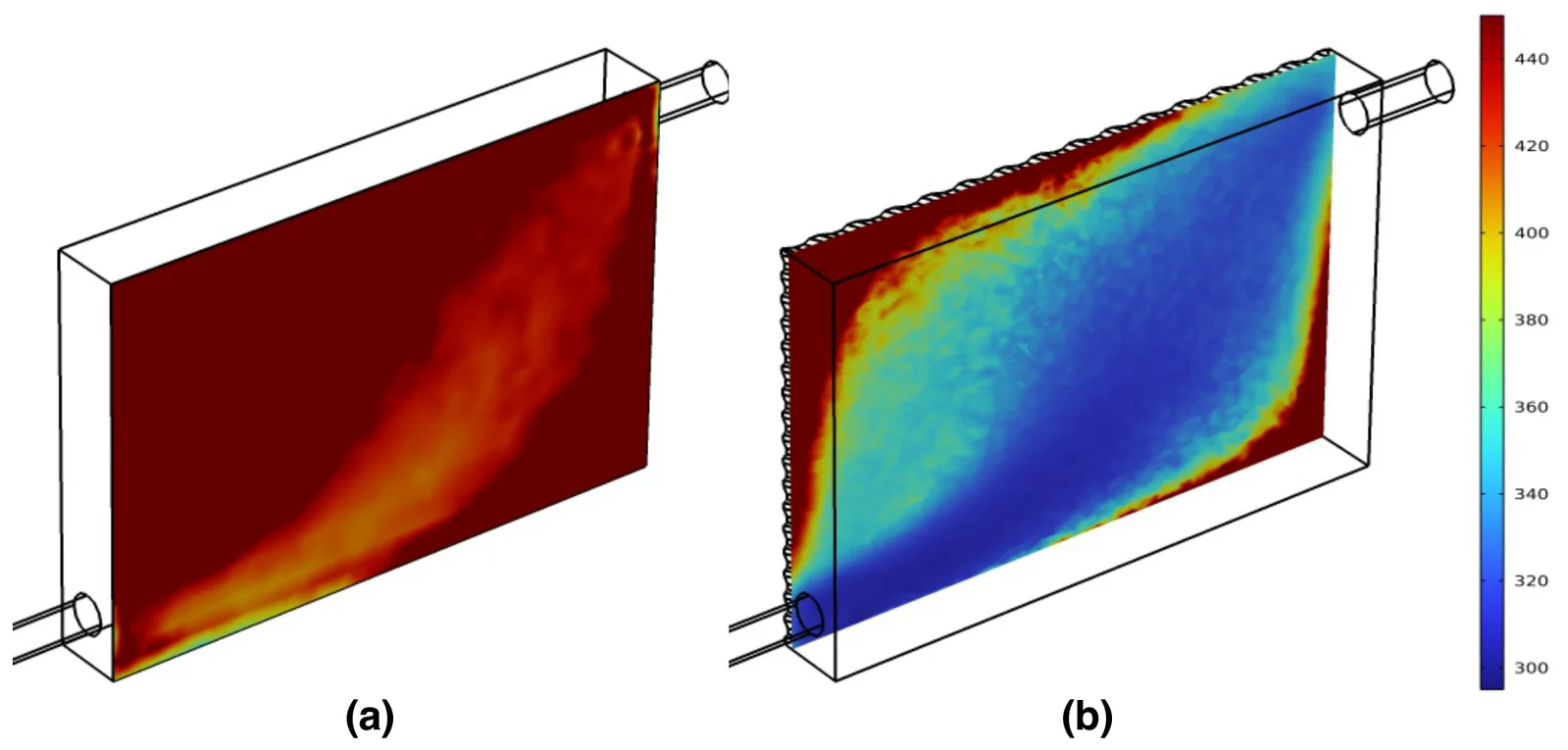
Steady-state distribution (after 4 min of operation) of $\bar{T}$ (K) in a fluid section in direct contact to active surface *A* for sunflower oil with $\dot{q}A=400$ W and $\dot{V}=1.00$ L/min). (**a**) Smooth *A* surface. (**b**) Modified *A* surface, per design modifications in Figure 7.

**Figure 9:**
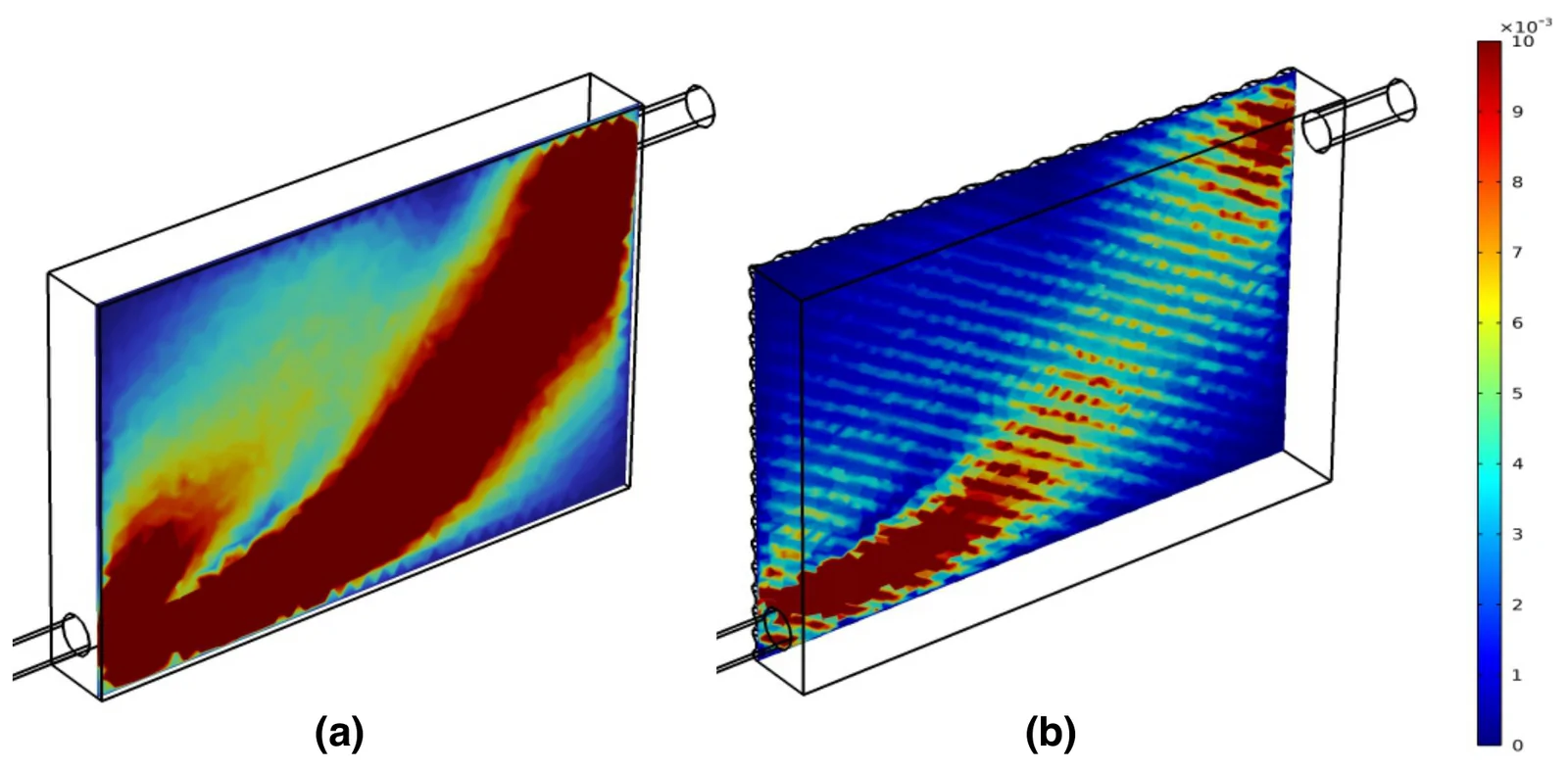
Steady-state distribution of $\left|\bar{v}\right|$ (m/s) in a fluid section in direct contact to active surface *A* for sunflower oil with $\dot{q}A=400$ W and $\dot{V}=1.00$ L/min). (**a**) Smooth *A* surface. (**b**) Modified *A* surface.

**Figure 10:**
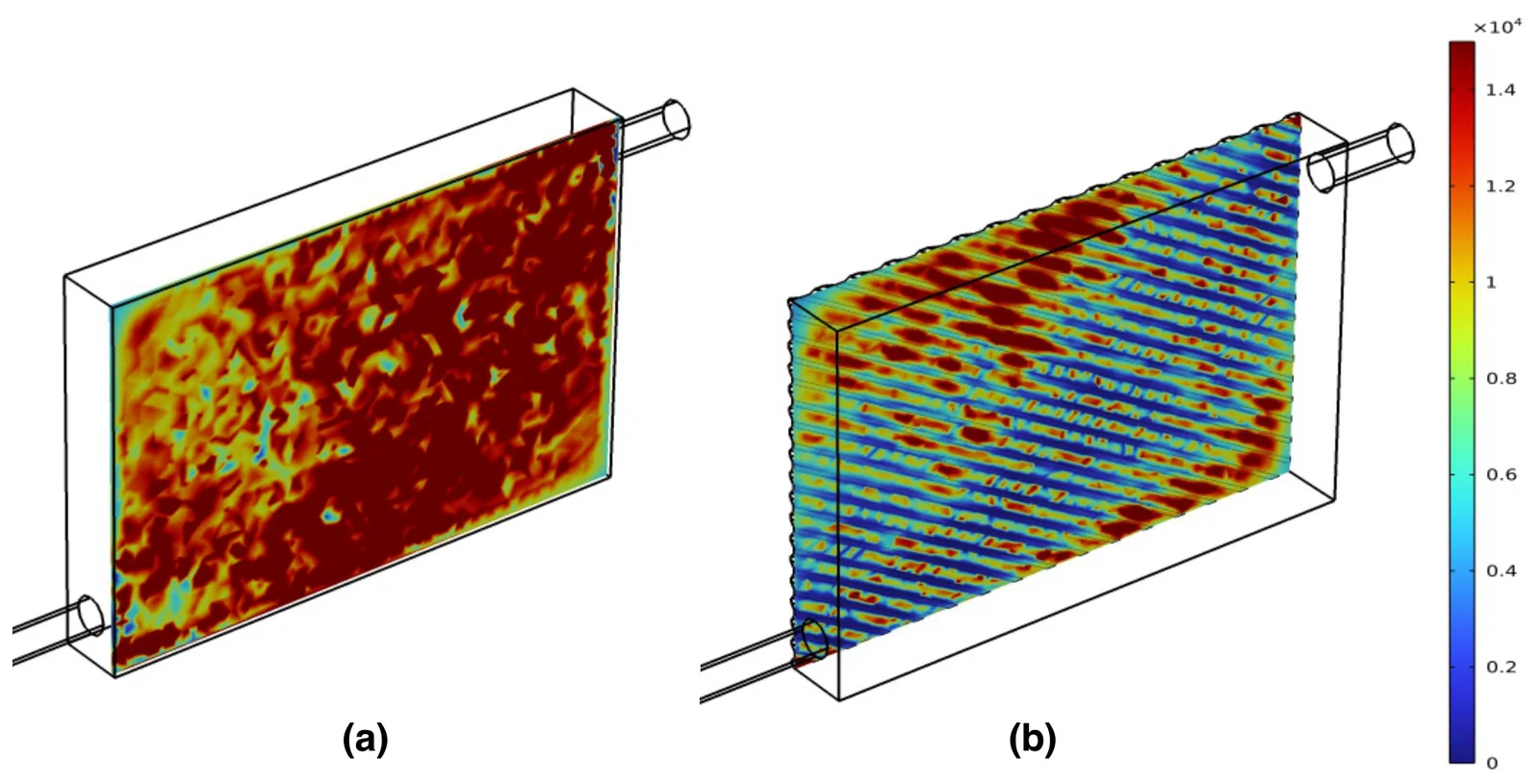
Steady-state distribution of ${\dot{S}}_{\mathrm{ht}}^{\prime \prime \prime }$ (W m^−3^K^−1^) in a fluid section in direct contact to active surface *A* for sunflower oil with $\dot{q}A=400$ W and $\dot{V}=1.00$ L/min). (**a**) Smooth *A* surface. (**b**) Modified *A* surface.

As a result, temperature and velocity maps can be altered considerably, as shown in Figure 8 and Figure 9 for the most severe regime in terms of entropy production, and with them the local entropy generation distributions, Figure 10.

**Figure 11:**
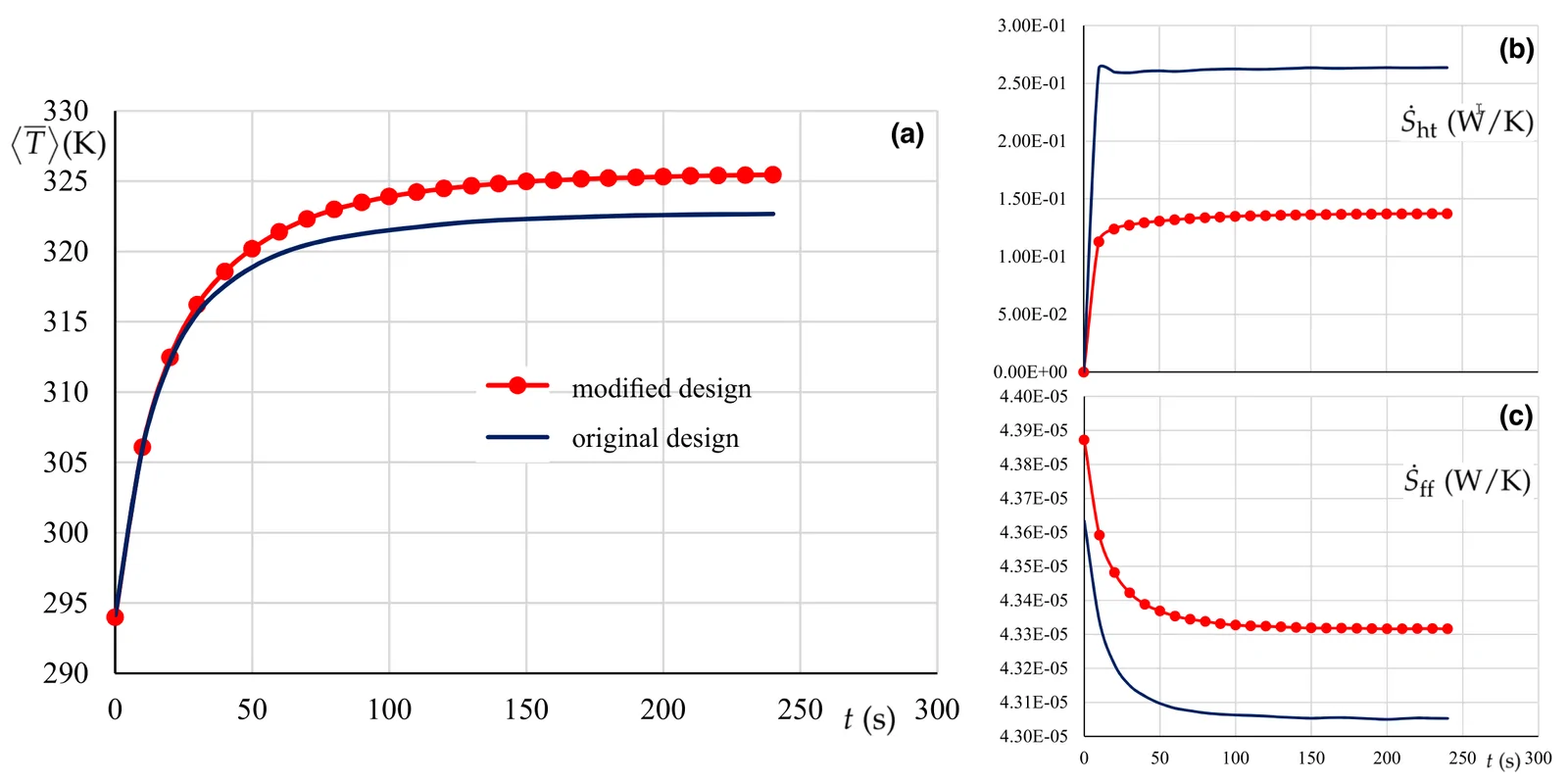
Progress of volumetric average $\langle \bar{T}\rangle$ (K) and ${\dot{S}}_{\mathrm{gen}}$ contributions (W/K) during the first 4 min of operation for sunflower oil with $\dot{q}A=400$ W and $\dot{V}=1.00$ L/min. (**a**) $\langle \bar{T}\rangle$. (**b**) ${\dot{S}}_{\mathrm{ht}}$. (**c**) ${\dot{S}}_{\mathrm{ff}}$.

In the same operating conditions, transient temperature and entropy generation contributions can be plotted, for the first 240 s of operation, as reported in Figure 11.

## 4. Discussion

Contrary to expectations, for a regular flow box as the present PFB-HEX design, no strong effect on local entropy generation distribution is found for the relatively small temperature and velocity gradients. Although large “hot spots” are found in low flow rate cases such as the one in Figure 3a where the thermal gradients in excess of 30 K are found (favored by a large-scale recirculation pattern in Figure 4a), local generated entropy leveling is seen for all cases, such as the one in Figure 5a; on the other hand, ${\dot{S}}_{\mathrm{ht}}$ is always about two orders of magnitude larger than ${\dot{S}}_{\mathrm{ff}}$ so that the Be number in Equation (13) is hardly less than 0.95, for any combination of the operating parameters, and can be dropped out for the scope of this analysis. Therefore we can focus on describing the $N_{\dot{S}}=f\left(\mathrm{Br}\right)$ relationship in Figure 6, based on the total entropy generation budget calculations.

Although entropy generation has frequently been correlated with the Brinkman number in the associated literature, a clear phenomenological interpretation linking Br to the physical mechanisms governing irreversibility redistribution has remained elusive. This gap motivated the present investigation, where the PFB-HEX with its simplest and most controlled process characteristics (as the applied power and flow rate) offered an ideal framework for entropy-resolved thermo-fluid analysis.

Generally, for both explored Prandtl numbers, the global entropy generation number $N_{\dot{S}}$ grows less than linearly with the applied power $\dot{q}A$, where only a slight decrease is found with the increasing flow $\dot{V}$, although a somewhat maximum entropy generation range exists for the highest applied power. Consequently, when seeking HEX optimization, the global entropy production incurred during the process is only marginally affected by the flow rate, while it increases gradually with higher thermal loads. For example, $N_{\dot{S}}$ for 2.00 L/min of sunflower oil warmed by 400 W, case (a) in Figure 6, is less than doubled with respect to case (b), when the power is just 150 W instead, while $N_{\dot{S}}$ decreases only slightly with increasing $\dot{V}$ from (b) to (c), with the aforementioned exception, in the (d)–(a) range. Even if limited to two fluid types only, it is believed that the presented chart contributes to highlighting the interconnection between entropy generation metrics and driving heat exchanger forcing parameters.

When a modified surface such as the one in Figure 7 is introduced in the flow box, local entropy generation maps can help rate the modified design. It is clear that the example presented here is not general in scope; it merely indicates possible directions for assessing a PFB-HEX from an entropy generation point of view. Under identical applied thermal power and flow rate, this mechanism enhances heat uptake by the flow while mitigating the formation of concentrated thermal gradients responsible for local irreversibilities: by comparing the two sides of Figure 8, it is indeed evident the merit of the modified design in keeping the active surface *A* colder, therefore transferring more heat directly to the flow. Repeated boundary-layer development across the modified surface, as seen comparing the two sides of Figure 9, substantially reduces the dominance of uniform localized thermal entropy production, which is evident by scrutinizing the two sides of Figure 10. The grooved ensemble acts as passive flow-guiding elements that improve thermal homogenization without requiring additional energy input, making the proposed arrangement particularly attractive for precision heating applications involving temperature-sensitive fluids.

In the end, the modified design promotes a higher thermal level of some 3 K in the first 4 min, which is a valuable ability when seeking optimized operation (Figure 11a). Indeed, the thermal contribution of entropy generation is almost halved (Figure 11b), at the expense of a modest fluid flow contribution, only.

Future developments will address topology optimization of the active surface and internal flow-guiding structures through entropy-generation minimization criteria. Further investigations will also consider non-Newtonian and multiphase sensitive fluids, to support the development of next-generation electricity-driven heat exchangers capable of achieving high thermal precision with minimal irreversible losses.

## Data Availability

No new data were created or analyzed in this study. Data sharing is not applicable to this article.

## Abbreviations

The following abbreviations are used in this manuscript:

CFD	Computational Fluid Dynamics
EGM	Entropy Generation Minimization
HEX	Heat EXchanger
PFB-HEX	Precision Flow Box Heat EXchanger
RANS	Reynolds-Averaged Navier–Stokes
